# Discriminating Clonotypes of Influenza A Virus Genes by Nanopore Sequencing

**DOI:** 10.3390/ijms221810069

**Published:** 2021-09-17

**Authors:** Ying Cao, Haizhou Liu, Yi Yan, Wenjun Liu, Di Liu, Jing Li

**Affiliations:** 1 National Virus Resource Center, Chinese Academy of Sciences, Wuhan 430071, China; caoyingor@163.com (Y.C.); liuhz@wh.iov.cn (H.L.); yanyi@wh.iov.cn (Y.Y.); 2 CAS Key Laboratory of Pathogenic Microbiology and Immunology, Institute of Microbiology, Chinese Academy of Sciences, Beijing 100101, China; liuwj@im.ac.cn; 3 State Key Laboratory for Conservation and Utilization of Subtropical Agro-Bioresources & Laboratory of Animal Infectious Diseases, College of Animal Sciences and Veterinary Medicine, Guangxi University, Nanning 530004, China; 4 Savaid Medical School, University of Chinese Academy of Sciences, Beijing 100039, China; 5 CAS Key Laboratory of Special Pathogens, Wuhan Institute of Virology, Center for Biosafety Mega-Science, Chinese Academy of Sciences, Wuhan 430071, China

**Keywords:** clonotypes, influenza A virus, nanopore, sequencing

## Abstract

Influenza viruses still pose a serious threat to humans, and we have not yet been able to effectively predict future pandemic strains and prepare vaccines in advance. One of the main reasons is the high genetic diversity of influenza viruses. We do not know the individual clonotypes of a virus population because some are the majority and others make up only a small fraction of the population. First-generation (FGS) and next-generation sequencing (NGS) technologies have inherent limitations that are unable to resolve a minority clonotype’s information in the virus population. Third-generation sequencing (TGS) technologies with ultra-long reads have the potential to solve this problem but have a high error rate. Here, we evaluated emerging direct RNA sequencing and cDNA sequencing with the MinION platform and established a novel approach that combines the high accuracy of Illumina sequencing technology and long reads of nanopore sequencing technology to resolve both variants and clonotypes of influenza virus. Furthermore, a new program was written to eliminate the effect of nanopore sequencing errors for the analysis of the results. By using this pipeline, we identified 47 clonotypes in our experiment. We conclude that this approach can quickly discriminate the clonotypes of virus genes, allowing researchers to understand virus adaptation and evolution at the population level.

## 1. Introduction

Influenza A viruses (IAV) are negative-sense, single-stranded, segmented RNA viruses with characteristics of high genetic diversity [1,2,3]. Wild birds are the natural reservoir of IAV, and bird migration can lead to IAV pandemics [4]. According to world health organization (WHO) estimates, the annual seasonal influenza can cause 5–10% of adults and 20–30% of children to become sick, with 3–5 million severe cases and 0.29–0.65 million deaths [5]. Additionally, the live poultry trade has contributed to the continued presence of avian influenza virus in China [6,7]. As a big breeding country, IAV research has much significance for public health. In addition, the endemic prevalence of H5N6, H7N9 and H9N2 subtypes of influenza viruses in poultry is a continuing threat to public health and farming enterprises [8,9,10]. The influenza virus genome consists of eight fragments. When viruses of different subtypes infect the same host, each fragment can be reassorted to generate new strains. The subtype of the influenza virus can be identified by knowing the genes coding surface glycoproteins [HA and NA] of the influenza virus, while other genes need to be defined as a clonotype. At this time, the virus composition in the host is complicated. Knowing the strain information can help us predict the dynamic changes of the virus. Studying each variation in a gene of IAV is complex and incomplete, and using a consensus sequence to represent all information of a viral population seems less accurate. It is obviously necessary to study the influenza virus from the perspective of individual clonotype. A clonotype refers to a set of single nucleotide variations (SNVs) found on the same genome. For influenza virus, we consider the combination of SNVs on each segment as a clonotype. By the way, viruses, even individual strains, are made up with a highly correlated but not identical and extremely large dynamic populations (which are often termed as viral quasispecies [11]).

With the increasing popularity of sequencing-based analysis methods, researchers and clinicians have begun to embrace this technology, especially (NGS). It is used as a method to detect unknown pathogens (such as the influenza virus [12,13], Ebola virus [14], and Zika virus [15,16]). NGS technologies that process millions of sequencing reads in one run provide an inexpensive, accurate, and fast diagnosis method for infectious diseases [17] and is considered extremely authoritative to study genetic variation in populations [18]. However, they are still restricted by short read output and are unable to discriminate individual clonotypes, and the FGS and NGS technologies are not suitable for clonotype studies of viruses.

TGS technologies, especially those represented by the MinION platform from Oxford Nanopore Technologies (ONT) and Pacific Biosciences RS II platform, have already been applied to genome assembly, epigenetic marker, transcriptomics, and metagenomics analyses of animal, plant, bacterial, and viral samples [19,20,21,22]. Compared to the PacBio RS II platform, the Oxford Nanopore MinION platform not only outputs longer reads (such as 2 M base-length) but is more handy and affordable. Although the Nanopore sequencing platform has been used to include real-time monitoring of Ebola virus [23], rapidly sequence influenza viruses during influenza epidemics and pandemics [24], and the origin and epidemic history of Zika virus [25]. The high error-rate [26,27] of the ONT technologies still limits the widespread application of the platform. Currently, the MinION platform has potential but is not feasible for the identification of different clonotypes in a virus population.

Herein, we evaluated the results of direct RNA sequencing and reverse transcribed cDNA sequencing of influenza viruses using the MinION platform. Further, we developed a novel algorithm to resolve the high error rate of single-molecule reads in MinION sequencing and the distribution of clonotypes in IAV. To the best of our knowledge, there are no related reports on the application of the MinION platform to the field of clonotype research of influenza genes. Therefore, this approach is a proof-of-concept study aiming to investigate the feasibility and superiority of using the MinION platform for discriminating virus clonotypes.

## 2. Results

### 2.1. Assembly and Statistical Analyses of Illumina Data

**Figure 1:**
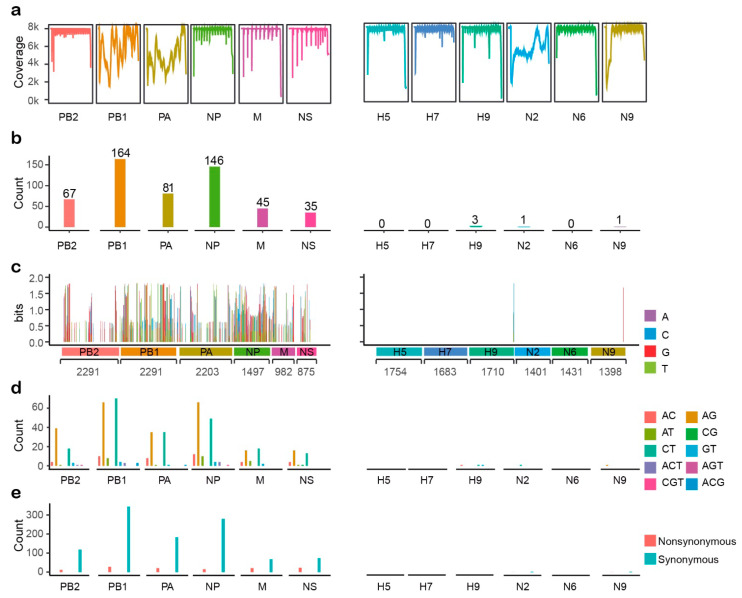
Statistical analyses of Illumina data. (**a**) Reads coverage for each segment of influenza A virus genome. (**b**) Number of SNPs in each segment. (**c**) Stack column plots shew variation distribution of each site along each segment. The column height depicts the information content, in bits. (**d**) The number of heterozygous base compositions in all variation sites for each segment. (**e**) The number of synonymous and nonsynonymous sites of each segment.

Influenza virus RNA extracted from allantoic fluid was reverse-transcribed into viral cDNA and sequenced using the Illumina HiSeq platform, producing 4.5 GB of data. De novo assembly and subsequent BLAST annotation identified 12 IAV-related complete segments: PB2, PB1, PA, H5, H7, H9, NP, N2, N6, N9, M, and NS, and the raw data for NGS sequencing were submitted to the Sequence Read Archive (SRA) database (accession number: SRR7496088). This result is consistent with that of the H5N6, H7N9, and H9N2 subtypes contained in the mixed infection samples determined by polymerase chain reaction using surface genes from different subtypes of influenza viruses. Illumina reads were mapped to assembly consensus sequences, and the coverage plots are presented in Figure 1a. The average sequencing depth was 7053-fold. In addition, variation calling of Illumina sequencing reads revealed that there were a total 543 variation sites in the poultry sample (Figure 1b), and the variation site numbers were highly diverse in different segments (Figure 1c). Most variation sites were identified in the internal genes of the influenza virus genome. On the contrary, there were few variation sites detected in the HA and NA segments in our sample. Furthermore, the base composition and nature of the variant sites were also explored. 531 of the total 543 variation sites only have two different bases, whereas another 12 sites were composed of three variant bases. Most of the variations were transitions, and AG and CT transitions occurred almost equally (Figure 1d). Moreover, 89.91% of the variations were synonymous, but in the M and NS segments, the proportion of nonsynonymous mutations was greater than in other segments (Figure 1e).

### 2.2. MinION RNA Sequencing of IAV Genes

**Figure 2:**
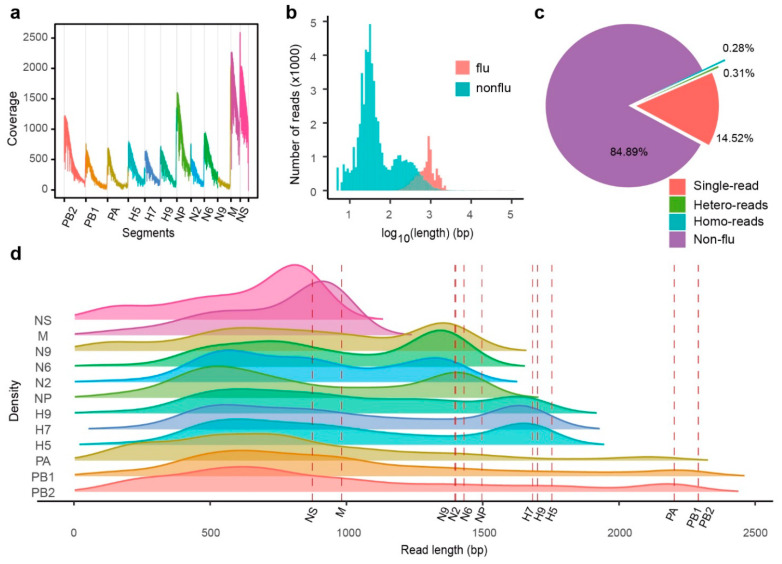
Statistics of direct RNA sequencing reads of MinION platform for influenza A virus sample. (**a**) Coverage of MinION direct RNA sequencing reads to Illumina consensus sequences. (**b**) Raw reads length distribution of MinION direct RNA sequencing reads, the flu reads (red) and the nonflu reads (blue). (**c**) Proportion of different kind of reads. Single-read was a raw read, containing only one segment. Hetero-reads were consisting of two different segments. Homo-reads were reads consisting of two same segments. Nonflu reads were not flu virus reads. (**d**) Density plot for length of each separated segment. The red dashed-lines depicted the length of each related segments, respectively.

Attempts to achieve full-length IAV genome sequences were performed using direct MinION RNA sequencing on total RNA extracted from allantoic fluid. After 24 h of sequencing, a total of 43 MB of data was collected, and 69,806 reads were generated. The maximum length of raw reads was 110,619 bp, and the median was 41 bp. Raw reads were mapped to the Illumina consensus sequences. All segments were completely covered, and the global average depth was 481-fold. The coverage of raw reads displayed a heavy bias toward the 3′ terminus (Figure 2a), similar to what Keller reported [22]. Annotation of raw reads was completed by BLASTN against the Illumina consensus sequences. The BLAST report indicated that 84.89% of the raw reads, including most short reads, were not similar to flu virus sequences (Figure 2b). For the remaining flu-similar reads, 14.52% were identified as containing only one viral sequence (single-read), whereas another 0.59% had two or more viral sequences (homo- and hetero-mixed read) (Figure 2c). All flu-similar reads were subsequently separated into flu-only sequences according to previous BLAST results. The distribution of the length of these sequences by density curves indicated that most sequences were much shorter than the known length of influenza A virus segments (the dashed lines), except the M- and NS-similar sequences (Figure 2d).

### 2.3. MinION cDNA Sequencing of IAV Genes

**Figure 3:**
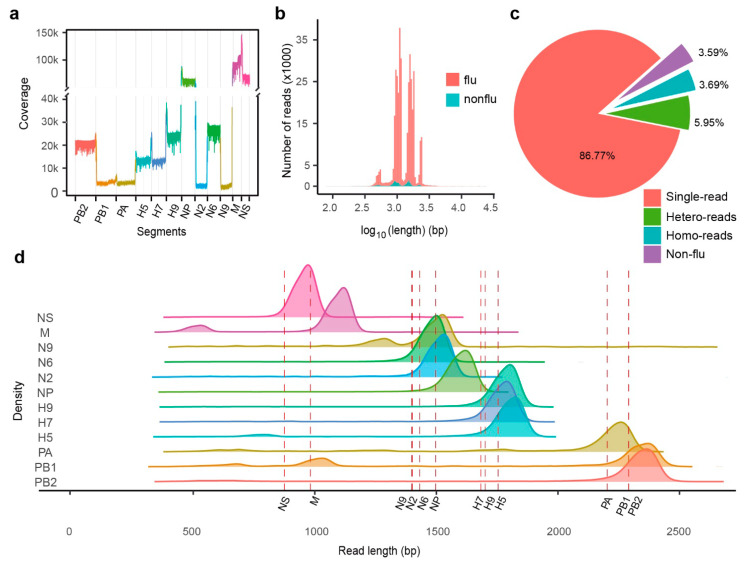
Statistics of cDNA sequencing reads of MinION platform for influenza A virus sample. (**a**) Coverage of cDNA sequencing reads of MinION platform to Illumina consensus sequences. (**b**) Length distribution of raw reads, the flu reads (red) and the non-flu reads (blue). (**c**) Proportion of different types of reads in raw data. Single-read was a raw read. containing only one segment. Hetero-reads were reads consisting of two different segments. Homo-reads were reads consisting of two same segments. Nonflu reads were not flu virus reads. (**d**) Density plots for length of separated segments. The red dashed lines depicted the length of each related segments, respectively.

Attempts to achieve full-length IAV genome sequences were performed via reverse transcription of total RNA extracted from allantoic fluid followed by cDNA sequencing using the MinION platform. Analogous to the direct RNA sequencing results, a similar analysis was performed on the cDNA sequencing results. MinION cDNA sequencing yielded 1.1 GB of data in a FASTQ file, and the sequencing experiment covered 100% of the influenza virus genome to an average depth of 26,567-fold (Figure 3a). In contrast to the RNA sequencing results, the length of most MinION cDNA sequencing reads was between 81 bp and 23.5 kbp, and the median was 1149 bp (Figure 3b). Further analysis of flu-similar reads indicated that mixed reads also existed. BLAST results of cDNA sequencing reads confirmed that ~10% of reads were mixed (homo- and hetero-mixed), and 3% were not flu virus reads (Figure 3c). Next, we separated mixed reads into singles according to the BLAST results (Figure 3d). We found that the length of most split sequences was close to the full length of each flu virus segment (labeled by dotted lines).

### 2.4. Comparison of Three Sequencing Strategies

**Figure 4:**
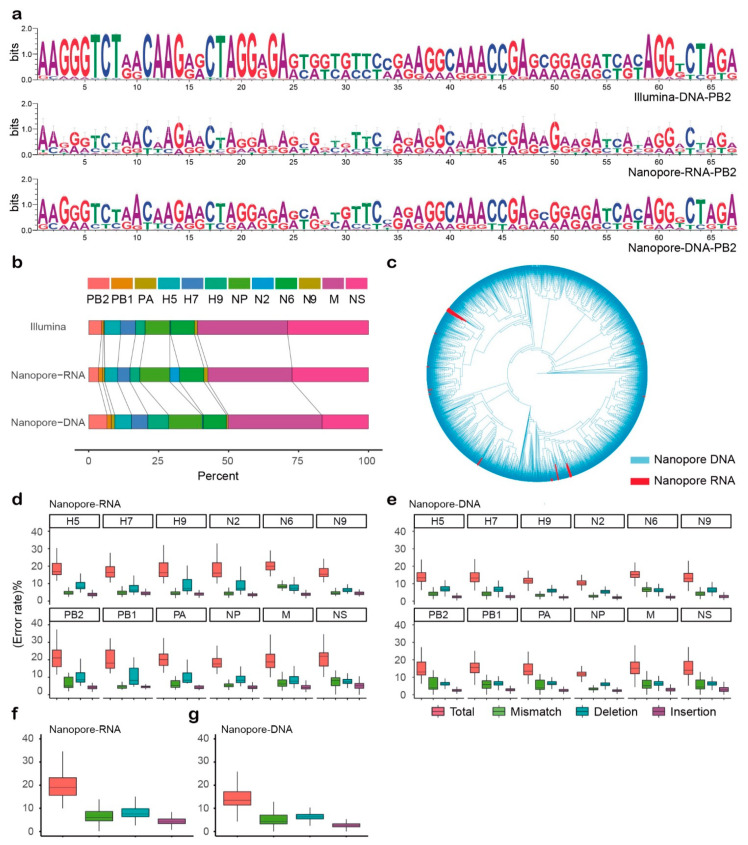
Comparison of sequencing methods among Illumina DNA and MinION RNA/cDNA. (**a**) Sequence logo for variation sites in three sequencing results. Variation sites were identified based on Illumina sequencing reads. (**b**) Number of separated reads for each segment in three sequencing methods. For Illumina sequencing result, the read number was the normalization of total mapped reads length over consensus sequence length for each segment, respectively. For MinION sequencing results, reads of with lengths greater than 80% of the segment full length were calculated. (**c**) Neighbor-joining tree of both direct RNA and cDNA sequencing reads included long reads (length ≥ 80%) only. Branch of direct RNA sequencing reads were colored in red. (**d**,**e**) Boxplots for total sequencing errors (mismatch and indels), and mismatch, indels, re-spectively for each segment in MinION direct RNA and cDNA sequencing. (**f**,**g**) Boxplots for total sequencing errors in MinION direct RNA and cDNA sequencing. Known variation identified by Illumina sequencing were not included. Outliers were not displayed for simplicity.

Next, we compared the differences between the two Nanopore MinION sequencing results with the Illumina sequencing results as the reference, and only long sequences (≥80% of the whole length) were analysed. The base composition at each variation site, which is represented by a Sequence Logo plot, implied that there were similar variation patterns and base compositions at these sites (Figure 4a and Supplementary Figure S1). Next, we found that the proportions of each segment in these sequencing results were also similar, except for the biases of the N2 segment in the Nanopore RNA sequencing results and NS in the Nanopore cDNA sequencing results (Figure 4b). This may have been caused by the RT-PCR and subsequent PCR amplification in the sample preparation step. However, the number of reads produced via direct RNA sequencing was much less than that produced via MinION cDNA sequencing. We also generated a tree by the neighbor-joining method of both MinION RNA and cDNA sequences. As can be seen from the data, although the sequence numbers of the two are different, their distribution is relatively uniform, which means the results of the two methods were comparable (Figure 4c). Finally, we compared the error rates between the two sequencing methods by the statistic of mismatches, deletions, and insertions (Figure 4d,e). In both sequencing results, there were more indels than mismatches, and the median of the total error rate in the direct RNA sequencing was ~19.56% (Figure 4f), which was higher than the 13.53% in the cDNA sequencing results (Figure 4g). The median of mismatch values of direct RNA sequencing was between 4.25% (N2) and 8.39% (N6), and the median of indel values varied from 10.69% (N9) to 13.02% (NS). For the cDNA sequencing, the mismatch median values ranged from 2.80% (N2) to 6.83% (N6), and median indel values were between 7.52% (N2) and 9.46% (NS). Therefore, the MinION cDNA sequencing results were more accurate than RNA sequencing. Considering that there were also much more available sequenced reads (≥80% whole segment length) and better accuracy, only cDNA sequencing data were used in the following analyses.

### 2.5. Clonotype Discrimination of IAV Internal Genes

**Figure 5:**
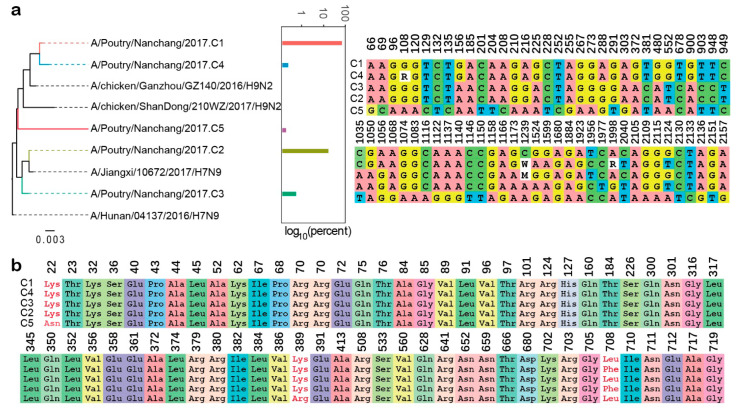
Phylogenetic analysis of influenza virus genome PB2 segment clonotypes. (**a**) Phylogenetic relationship, sequence abundance and sequence alignment of five clonotypes of PB2 segments. Numbers above alignment indicated the location of clonotype bases, and white background were redundant bases. (**b**) Alignments of corresponding amino acid residues of PB2 clonotypes.

Because our method worked on relatively long clonotypic sequences that were composed of >40 variation sites, we applied it to all internal segments of the poultry sample except NS. Doing so, 47 clonotypes were identified, including five of PB2, one of PB1, two of PA, three of NP, and 36 of M. We performed a phylogenetic analysis of the clonotypes of the PB2 gene and found that Clusters 1 and 4 were closely related to the H9N2 subtype, and Clusters 2, and 3 were similar to the H7N9 virus. The results of the sequence alignment were largely consistent with the topology of the ML tree, in which the base composition of Cluster 5 was different from the other clusters (Figure 5a). Alignments of a corresponding amino acid of PB2 revealed that most of the variations were synonymous mutations, which is consistent with the results of the Illumina sequencing (Figure 5b). The other clonotype cluster results are displayed in the Supplementary Materials (Supplementary Figure S2). The clonotypic sequence losses before and after the two-step clustering were calculated and are represented as neighbour-joining trees in Supplementary Figure S3. There were 81% and 42% clonotypic sequences of two major clusters remaining after clustering. This result also supports the credibility of this method.

### 2.6. Determination of Parameters for Clonotype Discrimination Presentation

**Figure 6:**
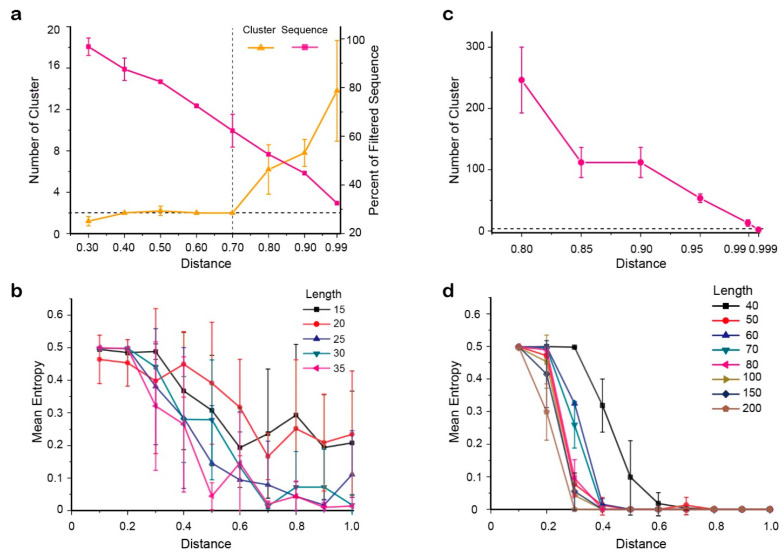
Simulation analyses for clustering thresholds identification and verification. (**a**) Identification of threshold I by five repeats with a fixed threshold II (=0.999). Dashed vertical line indicated the determined threshold I, 0.7. Horizontal line suggested 2 final clusters. (**b**) Confirmation of threshold II with a fixed threshold I (=0.7) by five repeats. Horizontal line suggested 2 final clusters. (**c**,**d**) Displayed the effect of clonotype length and the distance between the two clonotypes on the clustering results.

For the first clustering step, the threshold value in the second cluster step was fixed from 0.80 to 0.999, and distance values from 0.30 to 0.99 served for the first clustering step (Figure 6a). Considering both the number of generated clusters (smaller is better) and the proportion of removed one-sequence clusters (smaller is better), the final distance threshold value of the first cluster was 0.7. The distance threshold value for the second cluster was set to 0.999 after a series of tests with the first threshold value fixed at 0.7 (Figure 6b).

Due to the distance-based clustering method being sensitive to clonotype length, we applied this two-step cluster procedure to the different-length of clonotypic sequences (Figure 6c,d). The results indicated that the resolution of the method is positively correlated with the length of the clonotype sequence and the absolute distance between the two clonotypes, but it is not related to the product of these two factors. For instance, when the length of the clonotype (i.e., number of variation sites) was 40 bp, the two clonotypes could only be accurately distinguished when the two clonotypic sequences differed by 60% of the bases and the fault tolerance was 1.8%. Therefore, we concluded that the accuracy of this procedure is 98.2%. A different ratio of the two datasets was also simulated, and the results supported the robust nature of this procedure.

### 2.7. Comparison of Pre-Corrected and Modified Clonotype Sequences

**Figure 7:**
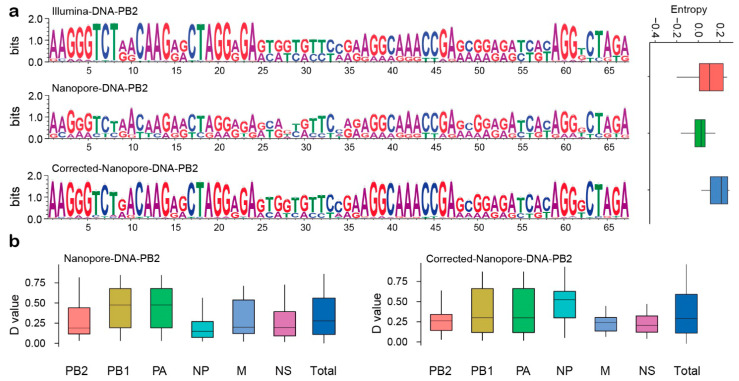
Demonstration of the effects before and after the modification of the clonotype sequences. (**a**) Sequence logo of variation sites and its entropy distribution in three sequencing results. (**b**) The distribution of the D value obtained by comparing the clonotype sequence of each fragment before and after modification with the clonotype sequence obtained by the Illumina platform.

In order to evaluate the efficiency of the simulation experiment, we calculated the correction efficiency of the simulation experiment and the effect of the experiment on the proportion of each gene were calculated. The base composition at each variation site, which is represented by a Sequence Logo plot, implied that there were similar variation patterns and base compositions between the corrected clonotype sequence and the results obtained via the Illumina sequencing platform (Figure 7a). Then, we used the effect size (*D* value) to evaluate the effect of the modified method of the simulation experiment on the proportion of each fragment in the genome. Because of the systematic differences between the Illumina sequencing platform and the MinION sequencing platform, the difference in base composition at each of the variant sites is inevitable. The box plot shows that the proportion of each segment did not change significantly before and after correction (Figure 7b).

## 3. Discussion

In this experiment, we chose mixed infected samples instead of individual strains as the research object. One reason is that poultry in live poultry markets come from different sources and live in complex environments, where they are highly likely to be infected by different subtypes of viruses. Another reason is that viruses are based on quasispecies rather than an individual. The Illumina sequencing results revealed that internal genes of the mixed infection sample have more variation than surface genes. A similar phenomenon was also found in related articles on H7N9 dynamic recombination [10], and the authors attributed this strange phenomenon to the use of the H9N2 internal gene cassette. The published data also showed that when an influenza virus has evolved within a host over a few seasons, the internal gene accumulates too much variation [10]. That is why it is far more difficult to study SNPs alone by NGS technology. The advantages of the ultra-long reads of the third-generation sequencing technology give us the possibility to study variation at the clonotype level.

We have evaluated direct RNA sequencing and cDNA sequencing with the MinION platform in terms of sequencing throughput, read length, and error rate. Our sequencing data shows that DNA sequencing is significantly better than RNA sequencing in length, throughput and error rate (Figure 2 and Figure 3). The higher proportion of deletions in the distribution of error types of the two sequencing methods compared to insertion and mismatches suggests that the primary reason for Nanopore platform, both RNA sequencing and DNA sequencing, is that the base perforation too fast and the existing algorithms cannot make effective distinctions between consecutive bases. It provides the direction for the new algorithm to correct the error rate of the Nanopore platform.

Since the direct RNA sequencing method is described [28], it has been used to study the genomes of influenza virus and coronaviruses [22,29]. Adrian et al. point out that direct RNA sequencing has the potential to reconstruct a large number of full-length clonotypes of RNA virus genomes, but further studies were not conducted. Based on our experiments, the RNA extracted from the mixed infection sample was directly sequenced using the MinION platform, referring to the method mentioned in the previous article [22]. Our study comprehensively sequenced RNA to identify mixed-infected samples and obtained accurate subtypes and near-full-length sequences. The segments generated by NGS were also detected by direct RNA sequencing, and the major components of the variant sites and the proportions of each segment were similar (Figure 4a,b). According to our results, the error rate of direct RNA sequencing (~19.56%) was too high to discriminate individual clonotypes. Further, the amount of data with long sequences was far from the requirements of subsequent analysis (≥80% whole length). Therefore, although there is no PCR bias or RT bias in direct RNA sequencing, the problems of high error rate and low-coverage must be addressed before it is useful for clonotype research.

Total RNA was reverse-transcribed into cDNA and sequenced with the Nanopore MinION platform. Whether in the proportion of each influenza segment or the composition of the variant sites, the results of cDNA sequencing were more similar to those obtained by Illumina sequencing than direct RNA sequencing. Based on the data obtained from DNA sequencing, we established a data analysis pipeline, and to correct the sequencing errors, the pipeline included two rounds of clustering. It is noteworthy that the reference sequence obtained by Illumina sequencing cannot be used to correct the reads of MinION sequencing due to viral genome diversity. The criteria of the two-round clustering were obtained by simulation of clustering of long and error-containing reads that were generated according to the error rate and distribution of error types of the Nanopore MinION sequencing technology. The size of the simulation dataset was the result of several tests, and the substantial simulation was fulfilled with a dataset composed with 10,000 random mutated sequences. In addition, the resolution of the two-step clustering procedure was clarified by two related datasets, of which the latter was generated from a mutated sequence originating from the seed sequence of the previous dataset. These two datasets were then combined into one for subsequent cluster analyses, and the ideal result would be that there were only two clusters left, with each cluster composed of sequences generated from only one seed sequence. The method can be used to quickly identify the clonotypes of virus genes, minimally more than 40 variation sites. And different simulation experiments on the proportion of the dataset show that as long as the number of datasets is large enough, the results are not affected by the data set proportions. In our pipeline, we corrected the cDNA sequencing data by simulating the process of Nanopore sequencing and improved the accuracy of clonotype identification to 98.2%. It was found that modification of the clonotype sequence by this method makes the base composition of the mutation site more similar to that obtained by Illumina sequencing.

In addition, due to the limitations of the clustering method, we could only distinguish clonotypes with a length > 40 bp, and clonotypes below this threshold may also be important. The method proposed in our study still provides a theoretical basis for research on non-primary clonotypes in viral populations, and the issue of the resolution of this method and its application will be the focus of our future attention.

The identification of different clonotypes in a population can help us to understand the changes of individual and determine the characteristics of clonotypes that are more adaptive to the environment. Moreover, the identification of clonotypes can also help to determine whether the virus causing a particular disease has the same clonotype, thereby linking the clonotype to the disease. Understanding the composition of clonotypes in viral populations can help lead to breakthroughs in medicine and biology. The identification of clonotypes allows us to both trace the source of the virus better and to provide a basis for studying the dynamic relationship between the clonotypes of different subtypes in the population and the environment.

To summarize, we expect that long read sequencing using the Nanopore platform will become increasingly valuable in the field of influenza virus genetics due to higher accuracy and the advantages mentioned above. The method we propose herein is simple, so we hope it will be a useful complement to the genome toolbox and may be generalized to the research of other viruses.

## 4. Material and Methods

### 4.1. Sample Screening and Preparation

Feces samples used in this study were collected with sterile cotton swabs from a live poultry market. Viruses were harvested from the allantoic fluid of 10-day-old SPF (Special Pathogen Free) chicken embryos according to the WHO manual (World Health Organization, 2002). The surface genes of different subtypes of influenza viruses were used for polymerase chain reaction (PCR) screening, and mixed infection samples were selected for further experiments. Total RNA was extracted with the NP968-C Nucleic Acid Extraction System and EX-RNA/DNA viral nucleic acid extraction kits (Tianlong Science and Technology Co., Ltd. Xi’an, China).

### 4.2. Illumina Library Preparation and Sequencing

The sequencing libraries were prepared via the following core steps: fragmenting the target sequences to 50–100 bp, end-repairing, attaching adenine oligonucleotide adapters to the ends of target fragments, PCR enrichment of adapter-ligated DNA, and quantitating the final library product for sequencing according to the manufacturer’s instructions (Illumina). Subsequently, the libraries with insert fragment lengths of 100 were sequenced on an Illumina HiSeq 2000 Sequencer.

### 4.3. Illumina Data Analysis

The raw Illumina reads were processed by removing low-quality reads, poly-Ns (>10 Ns), and adaptor-contaminated reads (>15 bp matched to the adapter sequence). The filtered reads were processed by de novo assembly using SOAPdenovo (version 1.06) [30] and Edena (version 3.121122) [31], respectively. The indels and mismatches were changed by aligning the de novo contigs (>200 bp) to the reference-based assembly sequences. Burrows-Wheeler Aligner (BWA version 0.7.15-r1140) [32] and SAMtools (version 1.3.1) [33] were then used to calculate sequencing coverage of the assembled consensus sequence of influenza virus genome. Site variation calling was performed using a Perl script as previously described [14] (Available at: http://github.com/generality/iSNV-calling/, accessed on 8 February 2020; note that a sequencing depth greater than 100 at a single locus is considered valid), and the assembled segments were used as the references for the analysis of the results of the Nanopore sequencing platform. A profile of all variation sites was then constructed. The distributions of variation sites, site heterogeneity, and synonymous and nonsynonymous substitutions were calculated by homemade scripts (Supplementary Figure S4a).

### 4.4. MinION RNA Library Preparation and Sequencing

Preparation of the direct RNA sequencing library for the MinION platform followed a previously published protocol [22]. It should be noted that total RNA was isolated using an Invitrogen PureLink Viral RNA/DNA Mini Kit (Thermo Fisher Scientific, Waltham, MA, USA) instead of Invitrogen TRIzol. The approximate experimental process was as follows: 200 μL of cell-free allantoic fluid was incubated at 56 °C for 15 min with 25 μL Proteinase K and 200 μL Lysis Buffer (containing 5.6 μg carrier RNA). Then, the above lysate was added to the Viral Spin Column in a collection tube and centrifuged at 6800× *g* for 1 min. Finally, RNA pellets were resuspended in 15 μL nuclease-free water after washing twice with 500 μL Wash Buffer (WII) with ethanol. In addition, the RNAs were quantified using a Quant-iTTM RiboGreen RNA Assay Kit.

The other steps were performed exactly as described: briefly, 500 ng of RNA was reacted with 1 μL adapter (RTA) for 10 min at room temperature, reverse transcription master mix was added to synthesize the first strand cDNA, and the reverse-transcribed RNA was connected to an RNA Adapter (RMX). Finally, the adapter-ligated RNA library was sequenced on the MinION platform using an FLO-MIN106 flowcell equipped with the R9.4 chemistry.

### 4.5. MinION cDNA Library Preparation and Sequencing

Preparation of a one-dimensional (1D) genomic DNA library was performed using the SQK-LSK108 system (Oxford Nanopore Technologies, Oxford, UK) according to the manufacturer’s instructions. First, viral cDNAs were synthesized from vRNAs by reverse transcription (RT) with the Uni12 and Uni13 primers [34]. Then, library preparation was as follows: 45 μL (total 1 μg) of end-repaired DNA was diluted to 60 μL by adding 7 μL ultra П End-prep reaction buffer, 3 μL ultra П End-prep enzyme mix, and 5 μL nuclease-free water. The sample was transferred to a 0.2-mL PCR tube and then incubated for 5 min at 20 °C and 5 min at 65 °C using a thermal cycler for end-repair. The end-repaired DNA was purified using 60 μL resuspended AMPure XP beads. A unique barcode was selected for every sample to run together on the same Flow Cell, and the reaction was incubated at room temperature for 10 min. Purification was achieved with 140 µL of Adapter Binding Buffer (ABB) twice using a magnetic stand, and the pre-sequencing mix was resuspended with 15 µL of Elution Buffer for 10 min in 37 °C. From this, the library that was used for loading into the MinION Flow Cell was prepared. Sequencing was performed for 48 h using flow cell version FLO-MIN107 (R9.5).

### 4.6. Analysis of MinION Data

Base calling of MinION Fast5 data was performed using Albacore v2.0.2 (Oxford Nanopore Technologies), and the output FASTQ format sequences were subsequently converted to FASTA format using FASTX-Toolkit v0.0.14. The coverage of the influenza virus genome was calculated by GraphMap (version 0.5.2) [35] and SAMtools. These reads were then identified by BLASTN (version 2.6.0) against the reference sequences provided by the NCBI Influenza Virus Sequence Annotation Tool [36], and some reads were found to contain more than one gene segment. Heterogeneous reads, which contained multiple gene segments, were then separated into single sequences using a self-made script and the length was calculated.

### 4.7. Comparison of Direct RNA and cDNA Sequencing

To compare the results of direct RNA and cDNA sequencing on the MinION platform, the sequence logo of mutation site, the proportion of influenza virus genes, and the error rate of sequencing were calculated. The sequence logos was drawn through the WebLogo 3 a web-based application [37]. When calculating the number of segments generated by the Illumina platform, the total number of reads was normalized by the length of each segment, because the reads produced by Illumina platform are smaller than the gene length. For the reads produced by the MinION platform, we calculated the length distribution of reads in order to ensure that sufficient data is retained and the effect of short reads on subsequent analysis is reduced. The reads with lengths < 80% of the complete gene were discarded, and each gene segment annotated split MinION reads was curated as a dataset.

Subsequently, the MinION sequencing errors, including insertions, deletions, and mismatches, were identified by BLASTN with assembled gene segments by Illumina consensus sequences as queries against the split MinION reads. Another homemade Perl script (all the script involved in the article is in https://github.com/zer0liu/OntClono, accessed on 8 February 2020) was used to calculate the proportion of mismatches and indels for each read. It is worth noting that the variation sites identified by Illumina sequencing were excluded for the calculation of MinION sequencing errors. Then, each read in the dataset was mapped to the consensus sequence, and only the base information of the sites in the variation site profile was kept. Lastly, each split MinION read was represented by bases in the variation sites and called the Phase I pre-clonotypic sequence (Supplementary Figure S4b). The phylogenetic trees of Phase I pre-clonotypic sequences (including readings from cDNA and RNA sequencing platform) were constructed by the neighbour-joining method in MEGA 7.0.26 [38].

### 4.8. Clonotype Analysis of Viral Genes

The procedure to determine viral gene clonotypes was fulfilled according to the flowchart presented in Supplementary Figure S4. The Phase I pre-clonotypic sequences with indels >10% were also discarded, and these led to the Phase II pre-clonotypic sequences. The threshold is based on statistical results of the distribution of insertions and deletions in all reads, which minimizes the impact of insertions and deletions on subsequent analysis while retaining most of the data information. The Phase II pre-clonotypic sequences were then subjected to clustering by CD-HIT [39]. The parameters of identity (-c) were determined by simulation (described below), and the word size parameter (-n) was the CD-HIT suggested value according to identity. After the first CD-HIT clustering, the resulting clusters with only one sequence were discarded. For the remaining clusters, alignment gaps in each member sequence of a cluster were replaced with the bases of the consensus sequence of the cluster. All corrected sequences were clustered by CD-HIT again, and the parameters of identity (-c) and word size (-n) were also determined by simulation (described below). The consensus sequence of each final cluster was designated as the clonotype of the viral gene.

### 4.9. Phylogenetic Analysis

Each base on the clonotype sequence of the virus gene was mapped to the corresponding position of the virus genome one by one to form a complete sequence by the homemade scripts. All available gene sequences that have a high identity with our viral genes were downloaded from the GenBank and the Global Initiative on Sharing Avian Influenza Data (GISAID) database. These sequences were aligned using Clustal Omega on the EBI web server [40]. Maximum likelihood (ML) phylogenies of all viral genes were estimated by RAxML-HPC2 on XSEDE [41], with GTR+Gamma model and a bootstrap value of 1000 selected.

### 4.10. Determination of Cluster Threshold by Simulation

To further shed light on the two CD-HIT cluster threshold values in the above procedure, we generated simulation datasets that mimicked the error rates of MinION output. Both CD-HIT cluster threshold values were determined using the simulation datasets. The key computational process of the simulation can be divided into the following four steps:

(1) The location of the variant site used to call the clonotype is random (simulate the steps in Supplementary Figure S4a).
(2) A sequence of 2000 bases in length was selected as the seed sequence of the original clonotype. A Perl script was used to generate a set of mutated sequences, of which the length was 2 kb, and imported random indels and mismatches according to the error rates of MinION results in previous analyses (simulate the steps in Supplementary Figure S4b). The clonotype dataset of these sequences was obtained by the same site variation calling script described above. For the selection of the dataset size, we generated simulation datasets for which the size varied (1000, 2000, 5000, 10,000, and 20,000), and calculate the each quartile, mean value of the matrix of the different clonotype datasets. The data size when the matrix eigenvalues tend to be stable was selected as the size of all data sets in the simulation experiment.
(3) Another related dataset was created to identify the resolution scope of this procedure. This new clonotype sequence dataset originated from a sequence that was similar to the starting sequence but with *n* random nucleotide mutations. These two clonotype datasets were then merged for the determination of the two CD-HIT cluster threshold values. In this dataset, sequences with gaps of >10% of the length were removed.
(4) The two cluster threshold values were independently determined by fixing the other one. To specify the first threshold value, the second distance value was fixed. The first distance value was set from 0.30–0.999 (Supplementary Figure S4c,d). The process of determining the second threshold is similar to the operation of the first threshold, and the second distance value is between 0.80 and 0.999 (including 0.80, 0.85, 0.90, 0.95, 0.99, and 0.999). (Here, the sequence identity threshold of CD-HIT (option -c) = 1 − distance value.)

Moreover, we simulated the different condition of mixed samples to learn the resolution of this method. Two simulation datasets, generated from different but identical length starting sequences, were merged into one dataset, with a total 20,000 sequences, but the ratio of the sequence numbers for these two datasets varied from 1:2, 1:10, 1:100, and 1:1000. Ultimately, we determined the applicability of this procedure for various clonotype lengths from 15 to 200. All of the above simulations were repeated five times, respectively.

### 4.11. Evaluation of the Effect and Bias of the Method

The correction efficiency of the simulation experiment and the effect of the experiment on the proportion of each gene were calculated. For the Phase II pre-clonotypic sequences before and after correction, the base composition of each mutation site was counted, and the sequence logos was drawn through the WebLogo 3 [37]. Next, the entropy of each mutation site was calculated and compared to the results obtained with the Illumina sequencing platform. Then, the effect size of each variant site before and after correction was calculated with reference to the base composition in each gene fragment of the influenza virus genome obtained from the Illumina sequencing platform (The effect size is a quantitative measure of the magnitude of a phenomenon, a larger absolute value always indicates a stronger effect). Finally, for the clonotypic sequences before and after the two-step clustering, the phylogenetic trees were constructed by the neighbor-joining method in MEGA 7.0.26 [38]

## Data Availability

All relevant data are included in this published article. The datasets used and/or analysed during the current study are available from the corresponding author on request. The raw data for NGS sequencing were submitted to the Sequence Read Archive (SRA) database (accession number: SRR7496088).

## Supplementary Materials

The following are available online at https://www.mdpi.com/article/10.3390/ijms221810069/s1. Figure S1. The sequence logo distribution of clonotypes from different sequencing platforms of PB2, PB1, PA, NP, M, and NS PA segment. Figure S2. Phylogenetic analysis of influenza virus genome PB1, PA, NP and M segment clonotypes. (a) and (b) Phylogenetic relationship, sequence abundance and sequence alignment of PB1, PA, NP, M and NS segments, respectively. Numbers above alignment indicated the location of clonotype bases, and white background were redundant bases. Alignments of corresponding amino acid residues of clonotypes. Figure S3. Difference of pre-clonotypic sequence Phase II and clonotypic cluster. Green branches were removed clonotypes in cluster process. Figure S4. Flowchart of operation with sample data for flu virus clonotypes. Ellipse indicated data and rectangle for operation processes. Illumina data, assembled sequences and variation sites, were served as the reference of Nanopore data. Pre-clonotypic sequences of Nanopore reads were then clustered twice by CD-HIT to obtain the final clonotype clusters.
