# Supplementary material for: Discriminating Clonotypes of Influenza A Virus Genes by Nanopore Sequencing
Source: Int J Mol Sci. 2021 Sep 17;22(18):10069. doi: 10.3390/ijms221810069 (PMC8468007; doi:10.3390/ijms221810069)
Supplement: Supplementary file 1 [file ijms-22-10069-s001.zip › ijms-1359642-supplementary.pdf]

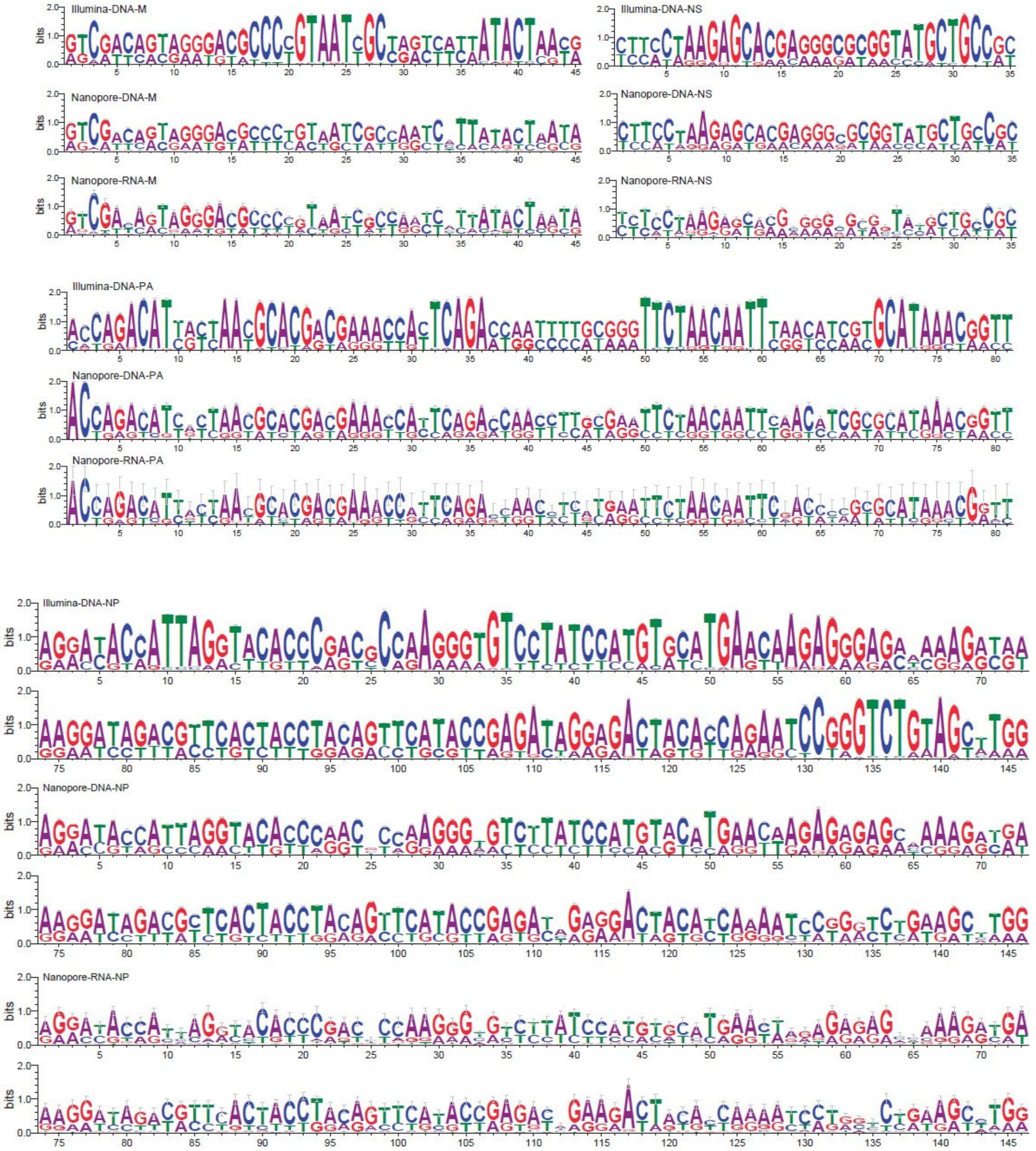

Figure S1. The sequence logo distribution of clonotypes from different sequencing platforms of PB2, PB1, PA, NP, M, and NS PA segment.

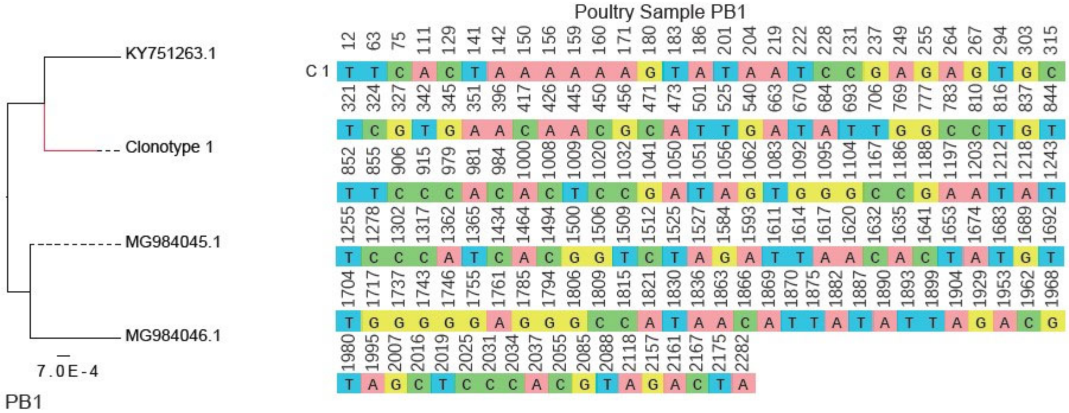

PB1

4 21 25 37 43 47 48 50 52 53 54 57 60 61 62 67 68 73 74 76 77 79 83 85 88 89 98 101 105 107 108 109 114 115
N T T G V H K S K G K T E T G N P L P D N P A T V L S G N C L E V Q

117 132 139 142 149 150 152 157 158 167 175 180 221 224 228 231 236 257 259 261 270 272 279 282 284 285 302 305 327 327 328 334 336 337
T T A A I F S A N L D E A L T A L A A S G P K L N V I D R R N R V L

340 344 347 350 351 352 354 361 364 365 368 389 396 396 399 401 404 406 415 419 426 434 439 454 455 478 488 498 500 502 503 504 509 509
A F K R L G G S L R V I L L G A S G L L L T D P N S G R G V A N L L

528 531 537 538 539 540 544 545 547 551 558 561 563 564 568 572 579 581 582 585 587 595 598 602 603 605 607 610 612 621 622 623 624 625
T K N D L G A Q A F T C R G I A L E Q S A G L R N H P C K Q G R L C

628 629 630 631 633 635 643 651 654 656 660 665 669 672 673 675 677 678 679 685 695 696 706 719 721 723 725
M N P F S K A P M E V S K R S L T S Q D L F R V R R D

PB1-F1

6 12 16 16 19 21 22 22 26 29 30 31 36 37 42 43 45 46 48 52 54 57
D S I I Q K E E Q R L E I Q Y L T T R H R Y

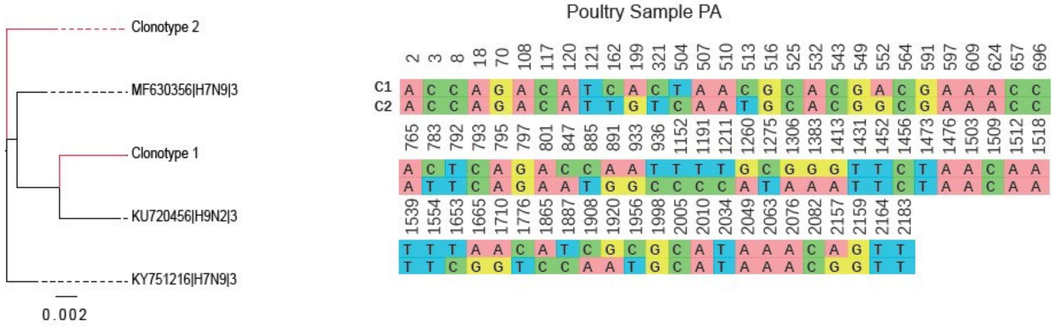

PA

2 20 32 35 36 37 50 63 103 164 165 166 167 168 171 174 177 179 180 184 193 195 199 204 215 228 251 257 260 261 261 262 263 279
E A T F A S D I P D E E S R I R T R Q S Q E E R A N K I F L L R T R
E A T F A S D V P D E E S R I R T R Q S Q E E R A N K I F L L R T R

291 293 307 308 380 393 400 416 421 432 457 467 473 480 482 487 488 497 499 500 502 509 514 547 551 566 588 618 625 632 636 648 662 665
S E A I D Y L E S V E T S F L S K K N L G S D D R R S K P S V S S L
S E A I D Y P E S I E T S F L S K K N L G S D D R R S T P S V S S L

666 674 679 684 688 690 715 716
L D G E E I L R
L D G E E I L R

PA-X

2 20 32 35 36 37 50 63 103 164 165 166 167 168 171 174 177 179 180 184 3 5 9 14 25 38 61
E A T F A S D I P D E E S R I R T R Q S M K K D P T K
E A T F A S D V P D E E S R I R T R Q S M K K D P T K

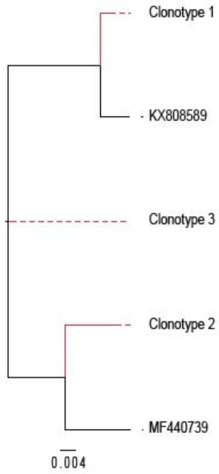

Poultry Sample NP

	51	54	78	81	87	100	102	105	114	132	150	154	165	174	180	186	204	231	234	235	254	255	258	264	267	291	292	294	297
C1	A	G	A	A	C	A	C	C	A	T	T	A	G	G	T	A	C	A	C	C	C	G	A	C	G	C	C	A	A
C2	R	A	G	A	T	A	C	A	A	T	T	A	G	A	T	Y	C	A	C	T	C	A	G	T	C	C	A	G	A
C3	G	A	G	A	T	A	C	A	A	T	T	A	G	A	K	T	C	A	Y	T	C	A	G	T	C	C	A	R	A

	306	339	354	363	369	375	384	396	405	414	420	426	438	441	444	453	471	474	480	484	495	498	504	507	513	540	549	555	556
	G	G	G	T	G	T	C	C	T	A	T	C	C	A	T	G	T	G	C	A	T	G	A	A	C	A	A	G	A
	A	A	A	A	G	T	T	T	T	T	T	C	T	Y	Y	G	T	A	T	C	T	G	A	G	Y	T	A	A	A
	G	A	A	W	G	T	T	T	T	T	T	C	T	M	T	G	T	R	C	N	T	G	A	G	T	T	A	A	A

	561	564	576	580	585	595	603	637	639	642	648	649	657	663	699	708	715	726	747	759	768	781	798	807	813	819	826	831	858
	G	G	G	A	G	A	A	A	A	A	G	G	C	G	A	A	G	A	A	A	C	A	G	T	C	G	C	T	C
	G	A	A	G	G	C	C	A	A	G	A	A	T	A	A	G	A	G	R	A	T	A	G	A	T	A	T	B	Y
	G	A	A	G	N	C	M	A	A	G	A	A	T	A	A	G	A	G	G	A	Y	A	G	A	Y	G	C	T	C

	864	870	873	885	891	892	897	900	945	951	960	972	978	982	984	1023	1026	1035	1041	1047	1054	1056	1068	1072	1080	1086	1092	1107	1117
	A	C	T	A	C	C	T	A	A	A	G	C	T	C	A	T	A	C	C	G	A	G	A	T	A	G	G	A	G
	A	C	T	A	C	T	G	A	C	A	G	T	Y	C	G	T	G	C	T	A	A	G	A	C	T	A	A	G	G
	A	C	T	A	C	C	T	A	A	A	G	C	T	C	A	T	A	C	C	G	A	G	A	T	A	G	G	A	G

	1119	1131	1134	1140	1141	1173	1176	1185	1194	1209	1212	1216	1218	1221	1230	1233	1239	1250	1251	1254	1260	1269	1284	1288	1302	1305	1314	1326	1349	1355
	A	C	T	A	C	A	T	C	A	G	A	A	T	C	C	G	G	G	T	C	T	G	T	A	G	C	T	T	G	G
	A	T	T	A	T	A	Y	C	A	A	A	G	C	Y	C	T	R	A	C	T	Y	A	A	A	G	T	A	K	G	G
	A	C	T	A	C	A	T	C	A	G	A	A	T	C	C	G	C	G	T	C	T	G	T	A	G	Y	T	T	G	G

17	18	26	27	29	34	34	35	38	44	50	52	55	58	60	62	68	77	78	79	85	85	86	88	89	97	98	98	99	102	113	118	121	123
G	E	R	A	V	S	S	G	R	C	S	N	R	Q	S	T	L	R	Y	L	A	A	G	D	P	Y	Y	R	R	G	K	R	R	A
G	E	R	A	V	S	S	G	R	C	S	N	R	Q	S	T	L	R	Y	L	A	A	G	D	P	Y	Y	R	R	G	K	R	R	A
G	E	R	A	V	S	S	G	R	C	S	N	R	Q	S	T	L	R	Y	L	A	A	G	D	P	Y	Y	R	R	G	K	R	R	A

125	128	132	135	138	140	142	146	147	148	151	157	158	160	162	165	166	168	169	171	180	183	185	186	187	188	192	194	195	199	201	213	213	214
N	D	G	H	I	H	N	A	T	Y	T	T	G	D	R	S	L	Q	G	T	G	V	G	I	G	T	E	I	R	R	I	R	R	R
N	D	G	H	I	H	N	A	T	Y	T	T	G	D	R	S	L	Q	G	T	G	V	G	I	G	T	E	V	R	R	I	R	R	R
N	D	G	H	I	H	N	A	T	Y	T	T	G	D	X	S	L	Q	G	T	G	V	G	I	G	T	E	V	R	R	I	R	R	R

216	217	219	221	233	236	239	242	249	253	256	261	266	269	271	273	276	277	286	288	290	291	295	297	298	299	300	315	317	320	324	326	328	328
R	V	Y	R	A	R	V	V	G	I	L	R	L	S	A	K	L	P	A	G	D	F	G	S	L	V	G	L	R	E	H	S	L	L
R	I	Y	R	A	R	M	V	G	I	L	R	L	S	A	K	L	P	A	G	D	F	G	S	L	V	G	L	R	E	H	S	L	L
R	I	Y	R	A	R	M	V	G	I	L	R	L	S	A	K	L	P	A	G	D	F	G	S	L	V	G	L	R	E	H	S	L	L

341	342	345	347	349	352	352	356	358	360	362	364	369	373	373	377	378	380	381	391	392	395	398	403	404	406	406	407	410	411	413	417	417	418
L	R	S	I	G	M	M	G	L	T	G	Q	E	A	A	N	T	E	L	R	S	N	Q	A	G	I	I	S	P	T	S	S	S	L
L	R	S	I	G	M	M	G	L	T	G	Q	E	A	A	N	T	E	L	R	S	N	Q	A	G	V	I	S	P	T	S	N	S	L
L	R	S	I	G	M	M	G	L	T	G	Q	E	A	A	N	T	E	L	R	S	N	Q	A	G	I	I	S	P	T	S	S	S	L

420	423	428	430	434	435	438	442	450	452
F	A	A	T	E	G	S	T	S	R
F	A	A	T	E	G	S	T	S	R
F	A	A	T	E	G	S	T	S	R

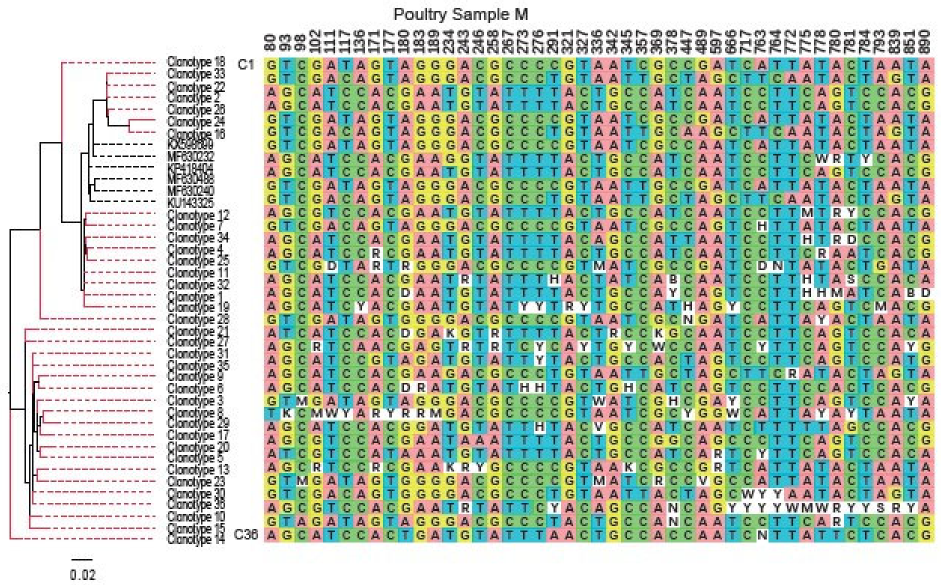

27	31	33	34	37	39	46	57	59	60	61	63	78	81	82	86	89	91	92	97	107	109	112	114	115	119	123	126	149	163	199	222	239
R	V	A	G	A	L	I	K	I	L	G	V	R	Q	N	G	D	N	N	V	M	F	A	E	V	Y	A	S	A	R	A	H	A
R	V	A	G	A	L	I	K	I	L	G	V	R	Q	N	G	D	N	N	V	M	F	A	E	V	Y	A	S	A	R	A	H	A
K	V	A	G	A	L	L	K	I	L	G	V	R	Q	N	G	D	N	N	V	I	F	A	E	V	Y	A	S	A	R	A	H	A
K	V	A	G	A	L	L	K	I	L	G	V	R	Q	N	G	D	N	N	V	I	F	A	E	V	Y	A	S	A	R	A	H	A
R	V	A	G	A	L	I	K	I	L	G	V	R	Q	N	G	D	N	N	V	M	F	A	E	V	Y	A	S	A	R	A	H	A
R	V	A	G	A	L	I	K	I	L	G	V	R	Q	N	G	D	N	N	V	M	F	A	E	V	Y	A	S	A	R	A	H	A
R	V	A	G	A	L	I	K	I	L	G	V	R	Q	N	G	D	N	N	V	M	F	A	E	V	Y	A	S	A	R	A	H	A
K	V	A	G	A	L	L	K	I	L	G	V	R	Q	N	G	D	N	N	V	I	F	A	E	V	Y	A	S	A	R	A	H	A
K	V	A	G	A	L	L	K	I	L	G	V	R	Q	N	G	D	N	N	V	I	F	A	E	V	Y	A	S	A	R	A	H	A
R	V	A	G	A	L	I	K	I	L	G	V	R	Q	N	G	D	N	N	V	M	F	A	E	V	Y	A	S	A	R	A	H	A
R	V	A	G	A	L	I	K	I	L	G	V	R	Q	N	G	D	N	N	V	M	F	A	E	V	Y	A	S	A	R	A	H	A
K	V	A	G	A	L	L	K	I	L	G	V	R	Q	N	G	D	N	N	V	I	F	A	E	V	Y	A	S	A	R	A	H	A
R	V	A	G	A	L	I	K	I	L	G	V	R	Q	N	G	D	N	N	V	M	F	A	E	V	Y	A	S	A	R	A	H	A
K	V	A	G	A	L	L	K	I	L	G	V	R	Q	N	G	D	N	N	V	I	F	A	E	V	Y	A	S	A	R	A	H	A
K	V	A	G	A	L	L	K	I	L	G	V	R	Q	N	G	D	N	N	V	I	F	A	E	V	Y	A	S	A	R	A	H	A
R	V	A	G	A	L	I	K	I	L	G	V	R	Q	N	G	D	N	N	V	M	F	A	E	V	Y	A	S	A	R	A	H	A
K	V	A	G	A	L	L	K	I	L	G	V	R	Q	N	G	D	N	N	V	I	F	A	E	V	Y	A	R/S	A	R	A	H	A
K	V	A	G	A	L	L	K	I	L	G	V	R	Q	N	G	D	N	N	V	I	F	A	E	V	Y	A	R/S	A	R	A	H	A
K	V	A	G	A	L	L	K	I	L	G	V	R	Q	N	G	D	N	N	V	M/I	F	A	E	V	Y	A	S	A	R	A	H	A
R	V	A	G	A	L	I	K	I	L	G	V	R	Q	N	G	D	N	N	V	M	F	A	E	V	Y	A	S	A	R	A	H	A
K	V	A	G	A	L	L	K	I	L	G	V	R	Q	N	G	D	N	N	V	I	F	A	E	V	Y	A	S	A	R	A	H	A
K	V	A	G	A	L	I	K	I	L	G	V	R	Q	N	G	D	N	N	V	I	F	A	E	V	Y	A	R	A	R	A	H	A
K	V	A	G	A	L	L	K	I	L	G	V	R	Q	N	G	D	N	N	V	I	F	A	E	V	Y	A	S	A	R	A	H	A
K	V	A	G	A	L	L	K	I	L	G	V	R	Q	N	G	D	N	N	V	M	F	A	E	V	Y	A	S	A	R	A	H	A
K	V	A	G	A	L	L	K	I	L	G	V	R	Q	N	G	D	N/K	N/K	V	I	F	A	E	V	Y	A	S	A	R	A	H	A
R	V	A	G	A	L	I	K	I	L	G	V	R	Q	N	G	D	N	N	V	M	F	A	E	V	Y	A	R/S	A	R	A	H	A
I	V	A	G	A	L	I	K	I	L	G	V	R	Q	N	G	D	N	N	V	M	F	A	E	V	Y	A	S	A	R	A	Q/H	A
K	V	A	G	A	L	L	K	I	L	G	V	R	Q	N	G	D	N	N/K	V	I	F	A	E	V	Y	A	S	A	R	A	H	A
K	V	A	G	A	L	L	K	I	L	G	V	R	Q	N	G	D	N	N	V	I	F	A	E	V	Y	A	R	A	R	A	H	A
K	V	A	G	A	L	L	K	I	L	G	V	R	Q	N	G	D	N	N	V	I	F	A	E	V	Y	A	S	A	R	A	H	A
K	V	A	G	A	L	L	K	I	L	G	V	R	Q	K	G	D	N	N	V	M	F	A	E	V	Y	A	S	A	R	A	H	A
R	V	A	G	A	L	I	K	I	L	G	V	R	Q	N	G	D	N	N	V	M	F	A	E	V	Y	A	S	A	R/S	A	H	A
R	V	A	G	A	L	I	K	I	L	G	V	R	Q	N	G	D	N	N	V	M	F	A	E	V	Y	A	S	A	R	A	H	A
K	V	A	G	A	L	L	K	I	L	G	V	R	Q	N	G	D	N	N	V	I	F	A	E	V	Y	A	R/S	A	R	A	H	A
R	V	E	G	A	L	I	K	I	L	G	V	R	Q	N	G	D	N	N	V	I	F	A	E	V	Y	A	R/S	A	R	A	H	A
K	V	A	G	A	L	L	K	I	L	G	V	R	Q	N	G	D	N	N	V	I	F	A	E	V	Y	A	S	A	R	A	H	A

Figure S2. Phylogenetic analysis of influenza virus genome PB1, PA, NP and M segment clonotypes. (a) and (b) Phylogenetic relationship, sequence abundance and sequence alignment of PB1, PA, NP, M and NS segments, respectively. Numbers above alignment indicated the location of clonotype bases, and white background were redundant bases. Alignments of corresponding amino acid residues of clonotypes.

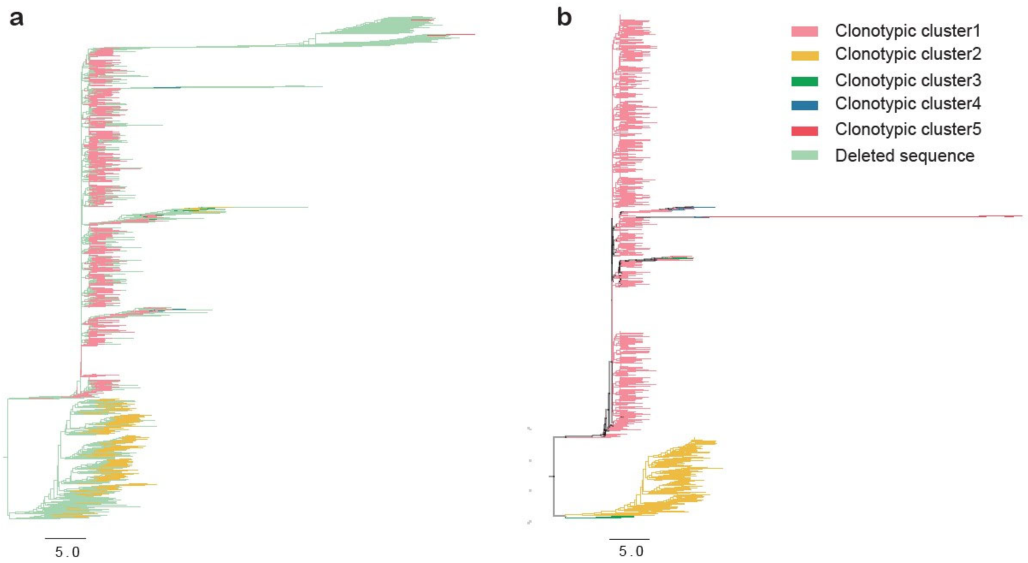

Figure S3. Difference of pre-clonotypic sequence Phase II and clonotypic cluster. Green branches were removed clonotypes in cluster process.

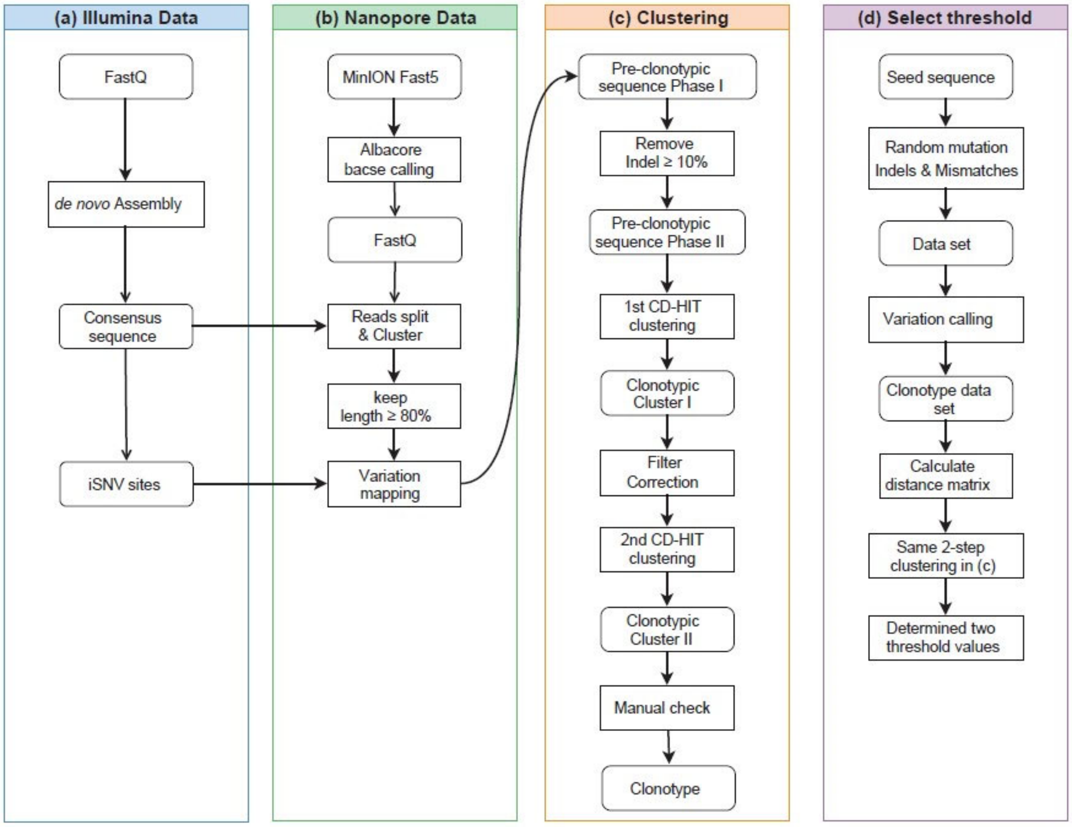

Figure S4. Flowchart of operation with sample data for flu virus clonotypes. Ellipse indicated data and rectangle for operation processes. Illumina data, assembled sequences and variation sites, were

served as the reference of Nanopore data. Pre-clonotypic sequences of Nanopore reads were then clustered twice by CD-HIT to obtain the final clonotype clusters.
